# Prognostic factors and outcomes of unplanned extubation

**DOI:** 10.1038/s41598-017-08867-1

**Published:** 2017-08-17

**Authors:** Chien-Ming Chao, Mei-I. Sung, Kuo-Chen Cheng, Chih-Cheng Lai, Khee-Siang Chan, Ai-Chin Cheng, Shu-Chen Hsing, Chin-Ming Chen

**Affiliations:** 1 Department of Intensive Care Medicine, Chi Mei Medical Center, Liouying, Taiwan; 2 Department of Internal Medicine, Chi Mei Medical Center, Tainan, Taiwan; 3 Department of Safety, Health, and Environmental Engineering, Chung Hwa University of Medical Technology, Tainan, Taiwan; 4 Department of Intensive Care Medicine, Chi Mei Medical Center, Tainan, Taiwan; 5 Department of Recreation and Health-Care Management, Chia Nan University of Pharmacy & Science, Tainan, Taiwan

## Abstract

This study investigated the prognostic factors and outcomes of unplanned extubation (UE) in patients in a medical center’s 6 intensive care units (ICUs) and calculated their mortality risk. We retrospectively reviewed the medical records of all adult patients in Chi Mei Medical Center who underwent UE between 2009 and 2015. During the study period, there were 305 episodes of UE in 295 ICU patients (men: 199 [67.5%]; mean age: 65.7 years; age range: 18–94 years). The mean Acute Physiology and Chronic Health Evaluation (APACHE) II score was 16.4, mean therapeutic intervention scoring system (TISS) score was 26.5, and mean Glasgow coma scale score was 10.4. One hundred thirty-six patients (46.1%) were re-intubated within 48 h. Forty-five died (mortality rate: 15.3%). Multivariate analyses showed 5 risk factors—respiratory rate, APACHE II score, uremia, liver cirrhosis, and weaning status—were independently associated with mortality. In conclusion, five risk factors including a high respiratory rate before UE, high APACHE II score, uremia, liver cirrhosis, and not in the process of being weaned—were associated with high mortality in patients who underwent UE.

## Introduction

Endotracheal intubation with mechanical ventilation (MV) support is an important intervention for managing patients with respiratory failure in the intensive care unit (ICU). After the respiratory condition has stabilized and the patient has been successfully weaned from MV, removal of the endotracheal tube removal (extubation) is scheduled. However, 2–16% of patients on MV undergo potentially life-threatening unplanned extubation (UE), which is defined as an accidental or a patient-induced removal of an endotracheal tube^1–6^. UE can cause several serious complications: aspiration pneumonia, bronchospasm, arrhythmia, respiratory failure, or even sudden cardiac arrest^7–10^. Furthermore, patients who undergo UE will have their MV, ICU stay, and hospital stay prolonged^6, 11–13^. The hospital mortality rate of patients who undergo UE can range from 10% to 25%, and even higher for patients who require reintubation^6, 12, 14, 15^. Although many studies^2, 8, 9, 11, 12, 16–19^ have mentioned UE-associated mortality, most of them focus on a comparison of mortality rates between patients who do and do not undergo UE. Some studies^8, 11, 16, 19^ have reported that UE is associated with a higher mortality rate, but others^9, 12, 17, 18^ have reported contrary findings. In the present study, we retrospectively reviewed the outcomes of patients who underwent UE in a medical center’s ICUs and calculated their mortality risk.

## Methods

### Study design and patient selection

A retrospective review of the medical records of all medical and surgical adult patients, including those in the 96 beds in Chi Mei’s 6 ICUs, who underwent UE between 2009 and 2015 was done in Chi Mei Medical Center. We calculated only the data of first UE for patients who underwent more than one UE during the same admission.

In our ICU, we would opiate agents including fentanyl or morphine as analgesia and benzodiazepines, including midazolam, lorazepam or propofol as sedative agents to keep patients being not agitated and comfortable according to the sedative protocol (appendix 1)^15^. Additionally, haloperidol can be prescribed for delirium and muscle relaxant including pancuronium or atracurium can be added if the above treatment failed. In addition, a weaning protocol was applied for the patients with acute respiratory failure ready for weaning (appendix 2). We also has set up a multidisciplinary team as physical therapist, respiratory therapist, critical care nurse and family to initiate the 4-level early mobilization program to improve the MV patients outcomes since April 1 2014^20^. In January 2014, the AnchorFast Guard tube fastener was applied to replace adhesive tape and cloth tape ties, as to well secure oral endotracheal tube. After the clinical condition improved and hemodynamic status became stable, we use pressure support ventilation for starting weaning and T-piece might be used. Extubation required acute weaning and the extubation protocol, or an order by an intensivist. Extubation was considered successful if reintubation was not required within the subsequent 48 hours. The reasons for re-intubating patients within 48 hours (failed extubation) included excessive secretions, acute respiratory distress with stridor, oxygenation failure, encephalopathy, and hemodynamic instability.

### Measurements

In this study, we collected the following data: (1) demographic and clinical variables: age, gender, cause of respiratory failure (cardiac, pulmonary, neurologic, gastrointestinal, hepatic, renal, and others), cause of reintubation, the duration of intubation before UE, and ICU type (medical or surgical); (2) disease severity: Acute Physiology and Chronic Health Evaluation (APACHE) II score, TISS score and GCS; (3) respiratory parameter and the result of laboratory examinations before extubation, such as respiratory rate (RR), the setting of ventilatior (positive end expiratory pressure [PEEP], tidal volume [TV] and etc.); and (4) outcomes: the UE rate (UE episodes per 100 ventilated patients), the failed UE rate (ratio of reintubation within 48 hours/100 UE), length of stay in ICU and hospital, total medical costs, and in-hospital mortality. The primary endpoint was factors that predicted mortality. The data were retrospectively collected and then analyzed. Therefore, informed consent was specifically waived and the study was approved by the Institutional Review Board of Chi Mei Medical Center (IRB: 10601-005).

### Statistical analyses

The comparison between non-survivors and survivors were examined using independent-samples *t* test for variables which were normally distributed and Mann-Whiney U test and χ^2^ test for those which were not. If *P* < 0.05, it was defined statistically significant. The variable with statistical significance in univariate analysis (*P* < 0.05), were further regarded as a focused risk factor of morality in the logistic regression model. SPSS 20.0 for Windows (SPSS, Inc., Chicago, IL, USA) was used for all analyses.

## Results

Between January 1, 2009, and December 31, 2015, there were 305 episodes of UE in 295 ICU patients. Because we only enrolled the first episode of UE for analysis, the first episodes of 295 patients were taken into further analysis. Forty-five patients died (mortality rate: 15.3%) (Table 1). One hundred ninety-nine patients (67.5%) were men (mean age = 65.7 years; age range = 18–94 years). The average APACHE II score was 16.4, TISS score was 26.5, and GCS score was 10.4. The average duration between time of intubation and UE was 134.4 ± 158.9 hours. One hundred thirty-six (46.1%) patients reintubated within 48 hours. Stroke (n = 93 [31.5%]) and diabetes mellitus (n = 73 [24.8%]) were the two most common comorbidities. Non-survivors had significantly higher APACHE II and TISS scores, medical originated patients, a higher reintubation rate, more comorbidities, and more uremia, liver cirrhosis, and cancer than did survivors.

**Table 1:** Demographic and clinical variables of the different unplanned extubation (UE) groups.

Item	All patients (n = 295)	Non-survivors (n = 45)	Survivors (n = 250)	*p* value
Age (years)	65.7 ± 16.1 (18~94)	68.0 ± 14.6	65.4 ± 16.3	0.317^a^
Female patients	96 (32.5%)	11 (24.4%)	85 (34.0%)	0.208
Body mass index	23.7 ± 4.5 (13.8~41.3)	24.0 ± 5.0	23.6 ± 4.4	0.591
APACHE II score	16.4 ± 8.7 (3~42)	22.9 ± 8.7	15.2 ± 8.1	<0.001
TISS	26.5 ± 9.1 (10~56)	30.3 ± 10.7	25.9 ± 8.6	0.015^a^
Glasgow Coma Scales	10.3 ± 4.1 (3~15)	9.4 ± 4.5	10.5 ± 4.0	0.129^a^
Intubation by pulmonary cause	112 (38.0%)	22 (48.9%)	90 (36.0%)	0.101
Time of MV before UE (h)	134.4 ± 158.9 (0~1231)	165.2 ± 239.7	128.8 ± 139.3	0.680^a^
Re-intubation within 48 h	136 (46.1%)	34 (75.6%)	102 (40.8%)	<0.001
Medical origin	154 (52.2%)	32 (71.1%)	122 (48.8%)	0.004
Endotracheal intubation	278 (94.2%)	45 (100%)	233 (93.2%)	0.118
Self-extubation	189 (64.1%)	27 (60.0%)	162 (64.8%)	0.537
Co-morbidity	1.0 ± 1.0	1.4 ± 1.1	1.0 ± 1.0	0.009
Coronary artery disease	71 (24.1%)	10 (22.2%)	61 (24.4%)	0.753
COPD	42 (14.2%)	6 (13.3%)	36 (14.4%)	0.850
Uremia	23 (7.8%)	12 (26.7%)	11 (4.4%)	<0.001
Liver cirrhosis	14 (4.7%)	9 (20.0%)	5 (2.0%)	<0.001
Diabetes	73 (24.7%)	16 (35.8%)	57 (22.8%)	0.068
Stroke	93 (31.5%)	10 (22.8%)	83 (33.2%)	0.145
Cancer	24 (8.1%)	8 (17.8%)	16 (6.4%)	0.017

Expressed as mean ± SD (range) or n (%); APACHE II = Acute Physiology and Chronic Health Evaluation; TISS = Therapeutic Intervention Score System; COPD = chronic obstructive pulmonary disease. ^a^The Mann-Whiney U test was used owing to not being normally distributed.

We compared the laboratory results before UE between the non-survivors and survivors (Table 2). Non-survivors had significant higher respiratory rates, higher FiO_2_ requirements, higher PEEP levels, and higher BUN and creatinine levels than did survivors. In contrast, survivors had significantly higher PaO_2_/FiO_2_, hemoglobin, hematocrit, and albumin levels than did non-survivors. Additionally, the timings of UE, the episodes of agitation, and need for and use of sedatives medications were not significantly different between survivors and non-survivors. Finally, non-survivors were prescribed and took lorazepam significantly more frequently than did survivors. Survivors also had a higher percentage of weaning stats (defined as the use of pressure support mode with a pressure level ≤14 cm H_2_O) than did non-survivors.

**Table 2:** Most recent data before extubation of different unplanned extubation (UE) groups.

Item	All patients (n = 295)	Non-survivors (n = 45)	Survivors (n = 250)	*p* value
Mean arterial pressure (mmHg)	98.0 ± 17.7 (50~153)	93.9 ± 23.0	98.8 ± 16.5	0.185
Heart rate (beats/min)	92.4 ± 17.1 (52~152)	95.0 ± 19.0	91.9 ± 16.7	0.365^a^
Respiratory rate (/min)	17.6 ± 5.7 (7~36)	19.3 ± 6.1	17.3 ± 5.5	0.026
Arterial blood gas data				
pH	7.43 ± 0.06 (7.13~7.59)	7.40 ± 0.09	7.44 ± 0.05	0.015
PaO_2_ (mmHg)	107.7 ± 56.1 (24~506)	104.6 ± 65.2	108.3 ± 54.5	0.695
FiO_2_ (%)	31.5 ± 12.7 (21~100)	36.8 ± 19.1	30.2 ± 10.2	0.029
PaO_2_/FiO_2_ (mmHg)	346.9 ± 131.9 (24~506)	313.8 ± 170.7	352.9 ± 123.0	0.152
PaCO_2_ (mmHg)	37.1 ± 8.3 (15.8~75.7)	36.7 ± 9.5	37.1 ± 8.1	0.802
Tidal volume (mL)	508.6 ± 152.1 (209~1458)	501.3 ± 132.3	510.0 ± 155.7	0.542^a^
PEEP (cmH_2_O)	5.9 ± 1.6 (0~14)	7.1 ± 2.2	5.6 ± 1.4	<0.001
Biochemistry data				
Hb	10.9 ± 2.0 (5.0~18.0)	10.2 ± 2.5	11.1 ± 1.8	0.012^a^
Hct	33.5 ± 7.0 (5.1~52.3)	31.0 ± 8.7	33.9 ± 6.6	0.013
BUN	31.3 ± 23.5 (3~163)	48.7 ± 35.3	28.1 ± 19.1	<0.001^a^
Creatinine	1.9 ± 2.5 (0.4~26.2)	2.7 ± 2.2	1.8 ± 2.5	0.024
Sodium	139.3 ± 6.3 (112.4~161.3)	138.6 ± 8.4	139.4 ± 5.9	0.542
Potassium	3.9 ± 0.5 (2.6~5.9)	3.9 ± 0.6	3.8 ± 0.5	0.713
Calcium	7.9 ± 0.9 (1.2~12.4)	8.0 ± 1.0	7.9 ± 0.9	0.579^a^
Phosphate	3.6 ± 1.8 (1.3~12.4)	4.2 ± 2.4	3.5 ± 1.6	0.292^a^
Albumin	2.7 ± 0.7 (0.9~4.4)	2.4 ± 0.7	2.8 ± 0.7	0.003
UE shift				0.234
Day time	102 (34.7%)	14 (31.3%)	88 (35.2%)	
Evening time	112 (38.0%)	14 (31.1%)	98 (39.2%)	
Night time	81 (27.5%)	17 (37.8%)	64 (25.6%)	
Agitation	95 (32.2%)	13 (28.9%)	82 (32.8%)	0.605
Sedatives	100 (33.9%)	17 (37.8%)	83 (33.2%)	0.550
Use of Lorazepine	24 (8.1%)	9 (20.0%)	15 (6.0%)	0.005
Weaning status	159 (53.9%)	12 (26.7%)	147 (58.8%)	<0.001

Expressed as mean ± SD (range) or n (%). ^a^The Mann-Whiney U test was used owing to not being normally distributed.

Non-survivors had non-significantly longer hospital stays, and higher hospital costs than did survivors. Non-survivors had significantly longer ICU stays than did survivors (Table 3). Multivariate analyses showed five risk factors—respiratory rate, APACHE II score, uremia, liver cirrhosis, and weaning stats—independently associated with mortality in UE patients (Table 4).

**Table 3:** Outcomes of different unplanned extubation groups.

Item	All patients (n = 295)	Non-survivors (n = 45)	survivors (n = 250)	*p* value
ICU stay (days)	14.7 ± 13.9 (1~127)	21.3 ± 21.7	13.5 ± 11.7	0.006^a^
Hospital stay (days)	36.1 ± 27.1 (1~206)	39.7 ± 37.6	35.5 ± 24.8	0.924^a^
Hospital cost (NTD, × 10^4^)	55.8 ± 45.7 (2.1~558.5)	67.9 ± 86.3	41.7 ± 35.9	0.004^a^

Expressed as mean ± SD (range); NTD = new Taiwan dollars. ^a^The Mann-Whiney U test was used owing to not being normally distributed.

**Table 4:** Mortality predictors of unplanned extubation using logistic regression model.

Variables	Odds ratio	95% confidence interval		*p* value
		Lower	Upper	
**APACHE II**	**1**.**058**	**1**.**006**	**1**.**114**	**0**.**028**
Comorbidity	1.247	0.855	1.821	0.252
**Uremia**	**5**.**811**	**1**.**566**	**21**.**559**	**0**.**009**
**Liver cirrhosis**	**5**.**920**	**1**.**463**	**23**.**96**	**0**.**013**
**Respiratory rate** (/**min**)	**1**.**092**	**1**.**014**	**1**.**176**	**0**.**020**
pH	0.030	0	19.166	0.288
FiO_2_ (%)	1.006	0.977	1.036	0.694
PEEP (cmH2O)	1.143	0.898	1.454	0.277
Hct	0.977	0.919	1.039	0.457
Creatinine	0.950	0.777	1.162	0.620
Albumin	0.685	0.351	1.337	0.268
**Weaning status**	**0**.**388**	**0**.**156**	**0**.**966**	**0**.**042**

n = 295

## Discussion

We found that a higher respiratory rate before UE, higher APACHE II score, uremia, liver cirrhosis, and not in the process of being weaned were independently associated with increased mortality in patients with UEs. This is consistent with the findings of other ICU studies. One Korean study^21^ reported the association between hospital mortality and reintubation, chronic neurological disease, emergency surgery, and higher APACHE II scores in UE patients in a surgical ICU. In addition, we found that a higher respiratory rate before extubation was associated with a higher risk of death in UE patients. This is because patients with tachypnea just before UE are not good candidates for extubation; thus, their mortality risk might be higher. In contrast, the mortality was significantly lower in UE patients being weaned before extubation. Our previous study showed that the rate of liberation form MV over 48 hours among UE patients were higher in those MV modes were setting in weaning condition (62.7%) as compared to those who were not (37.7%), and those successful weaning UE patients certainly had a significantly lower in-hospital mortality than failed UE patients (11.1% vs. 23.1%)^15^. Because these patients should be ready for extubation, if it occurs a little earlier than anticipated, it might not affect outcome. In addition, we has conducted an early mobilization program since April 1 2014^20^, and it may help to restore the muscle power during the critical phase, shorten the MV days and set patients ready for weaning. Those can partially help explain UE patients under weaning trial have a better outcome.

Uremia patients usually have multiple comorbidities and are prone to develop acute organ failure and in-hospital mortality. Our previous study displayed that the uremia patients had a significantly higher incidence of mortality than non-uremia patients among patients with acute respiratory failure requiring MV (342.30 vs. 179.67 per 1000 person-years, adjusted hazard ratio of 1.43)^22^. In this study, we have the similar finding that uremia was associated with higher risk of death among patients with UE.

Liver cirrhosis, in adults worldwide has become the 14^th^ most common cause of death, with an average year mortality of 1.03 million deaths^23^. In patients with decompensated liver cirrhosis, they would be accompanied with variceal bleeding, ascites, peritonitis, hepatorenal syndrome and hepatopulmonary syndrome. We previously showed that among critically ill patients with MV, those who with liver cirrhosis had more organ failures and had a significantly lower survival rate than non-cirrhosis controls (adjusted hazard ratio of 1.38)^24^. Similarly, the present study exhibited that cirrhosis could predict hospital mortality after the occurrence of UE.

More than 90% of our UE patients survived to discharge. This finding might imply that the extubation, planned and unplanned, of some patients in this study was delayed. Despite the weaning and extubation protocols of the six ICUs, the final decision to extubate could also be made by intensivists; therefore, it was possible to delay extubation. Finally, delayed extubation might have led to a relatively lower mortality rate in this study.

One recent review^25^ reported that reintubation rates range from 1.8% to 88% in UE patients. We found that 136 (46.1%) patients required reintubation within 48 hours after UE, and that the mortality rate of UE patients who required reintubation was higher than for patients who did not: 25.0% (34/136) vs. 6.9% (11/159). This is consistent with previous studies^14, 20, 26^. Overall, reintubation was associated with poorer outcomes in UE patients.

Other studies^25, 27, 28^ have reported several risk factors of UE: male gender, higher APACHE scores (≥17), chronic obstructive pulmonary disease, restlessness and agitation, lower sedation levels, higher consciousness levels, taking midazolam, and needing physical restraints. This is consistent with our findings on gender, the mean APACHE II score, agitation, and sedation.

This study had one major limitation: it was conducted in a single medical center. There might be differences about weaning and extubation decisions between our hospital and others. Therefore, our findings might not be generalizable to other hospitals. Additional large-scale studies are warranted to confirm our findings. In addition, we find that tachypnea prior to UE is a significant finding that predicts mortality. There are many reasons for tachypnea and since the paCO2 is not significant between groups, it is obviously not indicative of ventilation failure. Also, we did not show the tidal volume in ml/kg, as it might help to give an indicator as to their method of ventilation and determine if it is appropriate. Further survey is necessary to confirm these conclusions.

In conclusion, we found that a higher respiratory rate before UE, higher APACHE II score, uremia, liver cirrhosis, and not in the process of being weaned were risk factors associated with increased mortality in UE patients.

## Electronic supplementary material

Supplementary information
