# Supplementary material for: Prognostic factors and outcomes of unplanned extubation
Source: Sci Rep. 2017 Aug 17;7:8636. doi: 10.1038/s41598-017-08867-1 (PMC5561237; doi:10.1038/s41598-017-08867-1)

**Prognostic factors and outcomes of unplanned extubation**

**Running title:** Outcomes of unplanned extubation

Chien-Ming Chao, MD^1^, Mei-I Sung, RRT^2^, Kuo-Chen Cheng, MD^2,3^, Chih-Cheng Lai, MD^1^, Khee-Siang Chan, MD, PhD^4^, Ai-Chin Cheng, RRT^2^, Shu-Chen Hsing, RRT^2^, Chin-Ming Chen, MD^4,5,*^

^1^Department of Intensive Care Medicine, Chi Mei Medical Center, Liouying, Taiwan; ^2^Department of Internal Medicine, Chi Mei Medical Center, Tainan, Taiwan; ^3^Department of Safety, Health, and Environmental Engineering, Chung Hwa University of Medical Technology, Tainan, Taiwan; ^4^Department of Intensive Care Medicine, Chi Mei Medical Center, Tainan, Taiwan; ^5^Chia Nan University of Pharmacy & Science, Tainan, Taiwan

***Correspondence:** Chin-Ming Chen, MD, Department of Intensive Care Medicine, Chi Mei Medical Center, 901 Zhonghua Road, Yong-Kang District, Tainan City 710, Taiwan

**Tel:** 886-6-281-2811 Ext 57106; **Fax:** 886-6-282-8928; **Email:** chencm3383@yahoo.com.tw


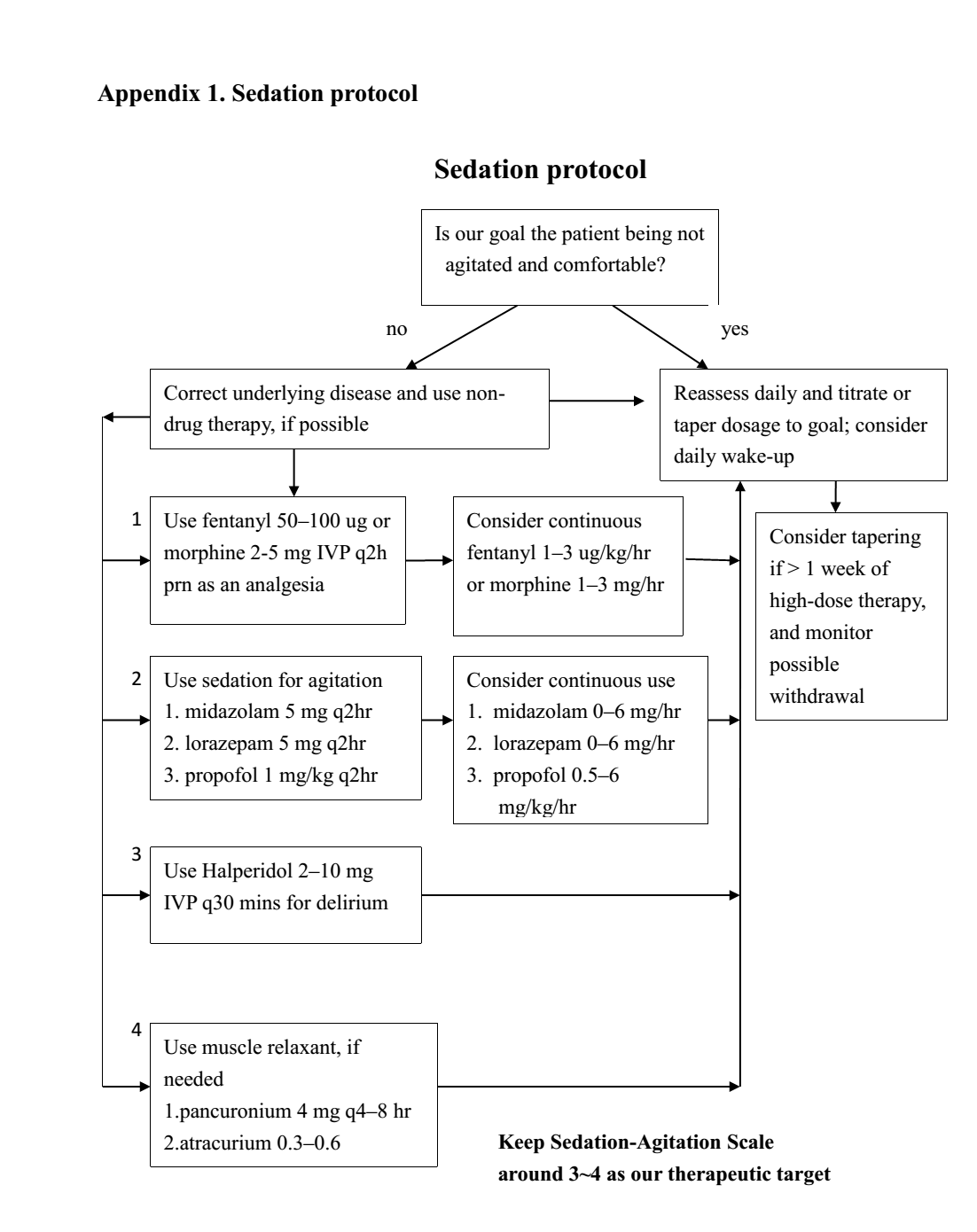


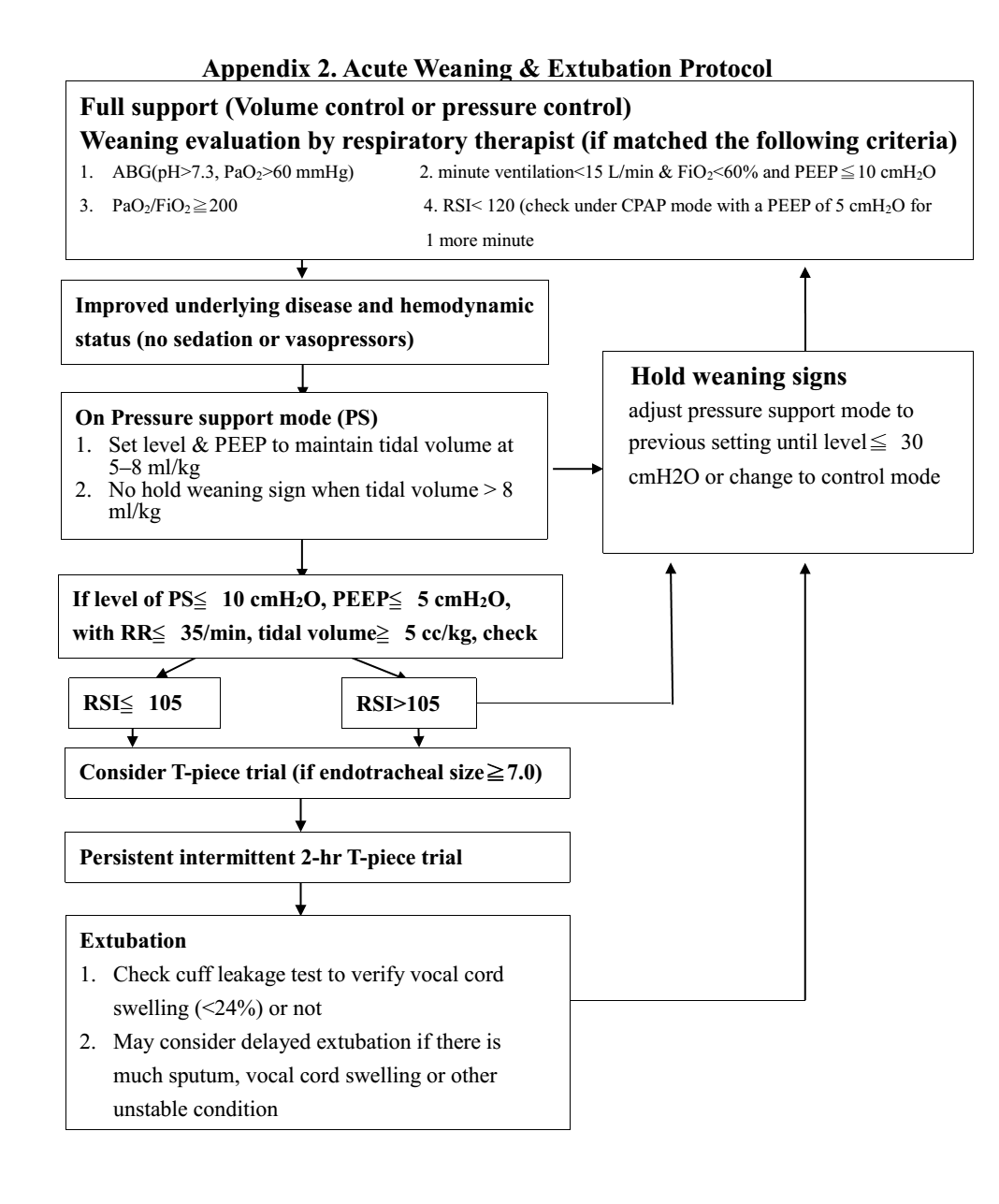

Supplement: Supplementary file 1 — Supplementary information [file 41598_2017_8867_MOESM1_ESM.doc]
